# Supplementary material for: Temporal assessment of N-cycle microbial functions in a tropical agricultural soil using gene co-occurrence networks
Source: PLoS One. 2023 Feb 14;18(2):e0281442. doi: 10.1371/journal.pone.0281442 (PMC9928094; doi:10.1371/journal.pone.0281442)
Supplement: S1 Checklist — (PDF) [file pone.0281442.s001.pdf]

Inclusivity in global research

PLOS' policy on inclusivity in global research aims to improve transparency in the reporting of research performed outside of researchers' own country or community and ensures that PLOS publications reporting global research adhere to high standards for research ethics and authorship. Authors of relevant research articles may be asked to complete the questionnaire below, which outlines ethical, cultural, and scientific considerations specific to inclusivity in global research. This questionnaire may be requested when researchers have travelled to a different country to conduct research, if research uses samples collected in another country, research with Indigenous populations or their lands, or if research is on cultural artefacts. Researchers travelling to another country solely to use laboratory equipment will not normally be required to complete the questionnaire. However, the questionnaire can be requested at the journal's discretion for any submission – if you have been requested to complete this questionnaire by the PLOS journal you submitted to, please do so.

Please complete the questionnaire below and include this as a Supporting Information file with your manuscript. Note that if your paper is accepted for publication, this checklist will be published with your article in the supporting information files. Please ensure that you reference the checklist in the main body of your manuscript. We suggest adding a subsection 'Inclusivity in global research' to your Methods section and adding the following sentence: "Additional information regarding the ethical, cultural, and scientific considerations specific to inclusivity in global research is included in the Supporting Information (SX Checklist)"

The questions have been designed to be applicable to a wide range of study types, and there are subsections for both human subjects research and non-human subjects research. If any of the questions are not relevant to your research please mark them as "N/A" as appropriate.

Ethical considerations, permits and authorship

This section is applicable to all research types.

Provide details as to who granted permissions and/or consent for the study to take place in the Methods section of your manuscript. This should include the names of **all** ethics boards, governmental organizations, community leaders or other bodies that provided approval for the study. If individuals provided approval refer to these people by their role or title but do not list their name(s).

Reported on page number: 8-9

If there were any deviations from the study protocol after approval was obtained please provide details of these changes in the Methods section of your manuscript.

Reported on page number: N/A

Did this study involve local collaborators that are residents of the country where the research was conducted or members of the community studied? If you do not have any authors from said communities, please provide an explanation for this below.

Yes, Dr. Mupenzi Mutimura is a co-author of the study and a resident of the country where samples were collected. As a senior scientist at the Rwanda Agriculture Board (RAB), Dr. Mutimura has a mandate to conduct agricultural research across Rwanda. Dr. Mupenzi Mutimura was instrumental to the creation and conception of the study design.

Everyone listed as an author should meet PLOS' criteria for authorship and all individuals who meet these criteria should be included in the author byline, rather than the acknowledgements. Authorship criteria is based on the International Committee of Medical Journal Editors (ICMJE) Uniform Requirements for Manuscripts Submitted to Biomedical Journals - for further information please see here: https://journals.plos.org/plosone/s/authorship.

Human subjects research (e.g. health research, medical research, cross-cultural psychology)

Did you obtain written informed consent from a representative of the local community or region before the research took place? How did you establish who speaks for the community? Details of written informed consent obtained from study participants should be reported separately in the Methods section of your manuscript.

N/A

How did members of the local community provide input on the aims of the research investigation, its methodology, and its anticipated outcome(s)?

N/A. Research was performed on agricultural research stations, in collaboration with the Rwandan Agricultural Board (RAB).

When engaging with the local community, how did you ensure that the informed consent documents and other materials could be understood by local stakeholders?

N/A. Research was not performed on human subjects.

Will the findings of the research be made available in an understandable format to stakeholders in the community where the study was conducted (e.g. via a presentation, summary report, copies of publications, etc.)? Please provide details of how this will be achieved.

N/A. Not human research. Although this project did not engage directly with human subjects, results have been disseminated to local farmer communities in the vicinity of the research stations where soil samples were collected. Approximately 40 households received large print, laminated posters containing extension infographics translated into the local language (Kinyarwanda). The infographics translated study findings into locally appropriate forage management recommendations, within RAB's mandate to conduct agricultural research and convey findings to Rwandan farmers. A RAB field technician disseminated the results within each community.

Non-human subjects research using specimens/ animals collected as part of the study, or those housed in archival collections. Examples include archaeology, paleontology, botany and zoology.

Did the permission you obtained from a local authority to perform the study include an agreement on access to outputs and benefit sharing? This may include procedures to enable fair distribution of the benefits and resources arising from the research performed. Please include any details of Prior Informed Consent and Benefit Sharing Agreements obtained. These may be required by field-specific regulations, for example the Convention on Biological Diversity (CBD) and the associated Nagoya Protocol.

Yes, this study falls under an agreement between the Rwanda Agriculture Board and the International Center for Tropical Agriculture. The agreement stipulates:

1. Each party is and remains the sole owner of all background intellectual property developed prior to this agreement or acquired or developed parallell to said agreement without being related to its execution. Background intellectual property includes any information, techniques, know-how, practices, processes, testing procedures, software and materials that is known to either party before the project and that the parties might exchange in relation to the project.
2. All tangible and intangible results that arise from this project shall be international public goods.
3. The results of the research will be jointly owned by the parties, and should be publishable in accordance with normal academic practice, with emphasis in the use of suitable open licences and Open Acess journals and publications, and the parties agree that researchers engaged in the project shall be permitted to make oral and written submissions at symposia, national, or regional professional meetings, and to publish in journals, theses or dissertations.
4. Best efforts shall be used by the parties to make all information products Open Access through suitable Open Access repositories, in the timeframe suitable for the type of information product, as stated in the CGIAR Open Access Policy which is an integral part of this agreement.

If the material used in your study was imported, please A) provide the year it was imported and B) indicate whether permits were obtained to import/export the materials used, C) provide details of any permits obtained. If this information is not available, please indicate this.

Soil samples were imported from Rwanda to the United States between September 2020 – February 2021. International soil samples were imported under a valid USDA-APHIS permit (P330-21-00156). Permission to export soil from Rwanda was also obtained from the Rwanda Ministry of Agriculture and Animal Resources Office of Agriculture and Livestock Inspection and Certification Services. The Office of Agriculture and Livestock Inspection and Certification Services issued a new export agreement for each shipping event which did not contain a unique identification number. Approval for the export permits was given by an authorized agent of the Rwanda National Plant Protection Organization.

If you used archival specimens, please state how the material used in your study was acquired by the institute it is held in and provide details of any permits obtained for the original excavations/ sample collection. If this information is not available, please indicate this.

N/A.

How was the potential cultural significance of the materials collected in your study to local communities considered in your research design? Were Indigenous peoples and/or local researchers and institutions involved with archaeological excavations / collection of specimens? If so, please provide a description of their involvement.

We worked closesly with research scientists and field staff at the Rwandan Agricultural Board to collect soil samples, and who approved of the study design. Local Rwandan technicians worked exlusively on sample collection and plot maintenance.

If your manuscript includes photographs of human remains please indicate whether authors obtained permission from descendants or affiliated cultural communities to do so.

N/A.
