# Supplementary figures and images for: Temporal assessment of N-cycle microbial functions in a tropical agricultural soil using gene co-occurrence networks
Source: PLoS One. 2023 Feb 14;18(2):e0281442. doi: 10.1371/journal.pone.0281442 (PMC9928094; doi:10.1371/journal.pone.0281442)

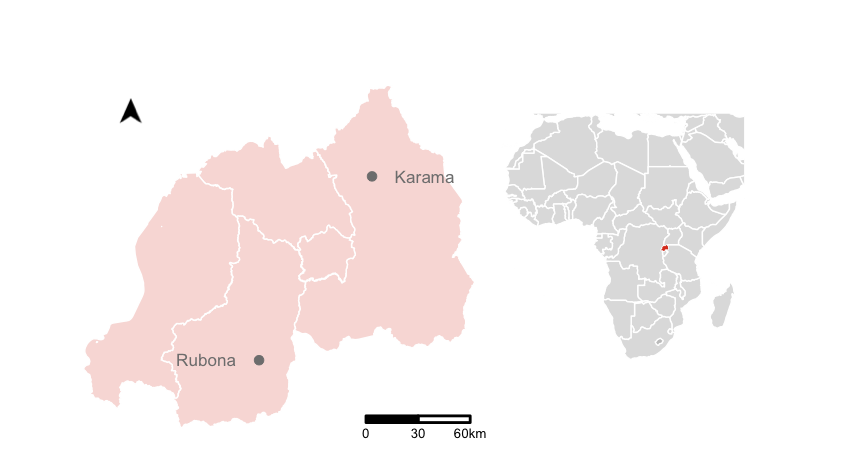

Supplement: S1 Fig — This map was generated using the ‘maps’ package in R, which imports data from the public domain Natural Earth project. (TIFF) [file pone.0281442.s002.tiff]

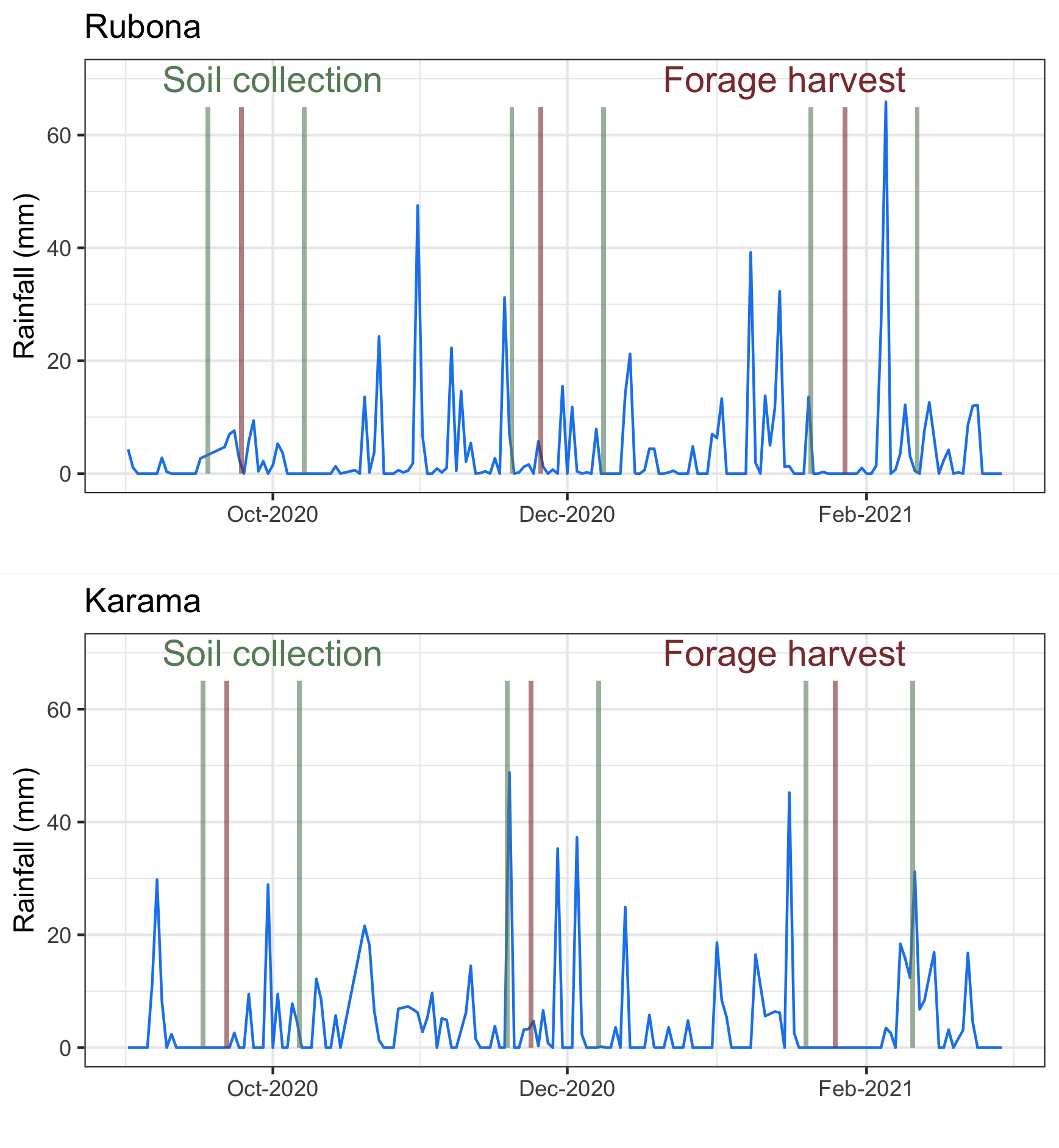

Supplement: S2 Fig — (TIFF) [file pone.0281442.s003.tiff]

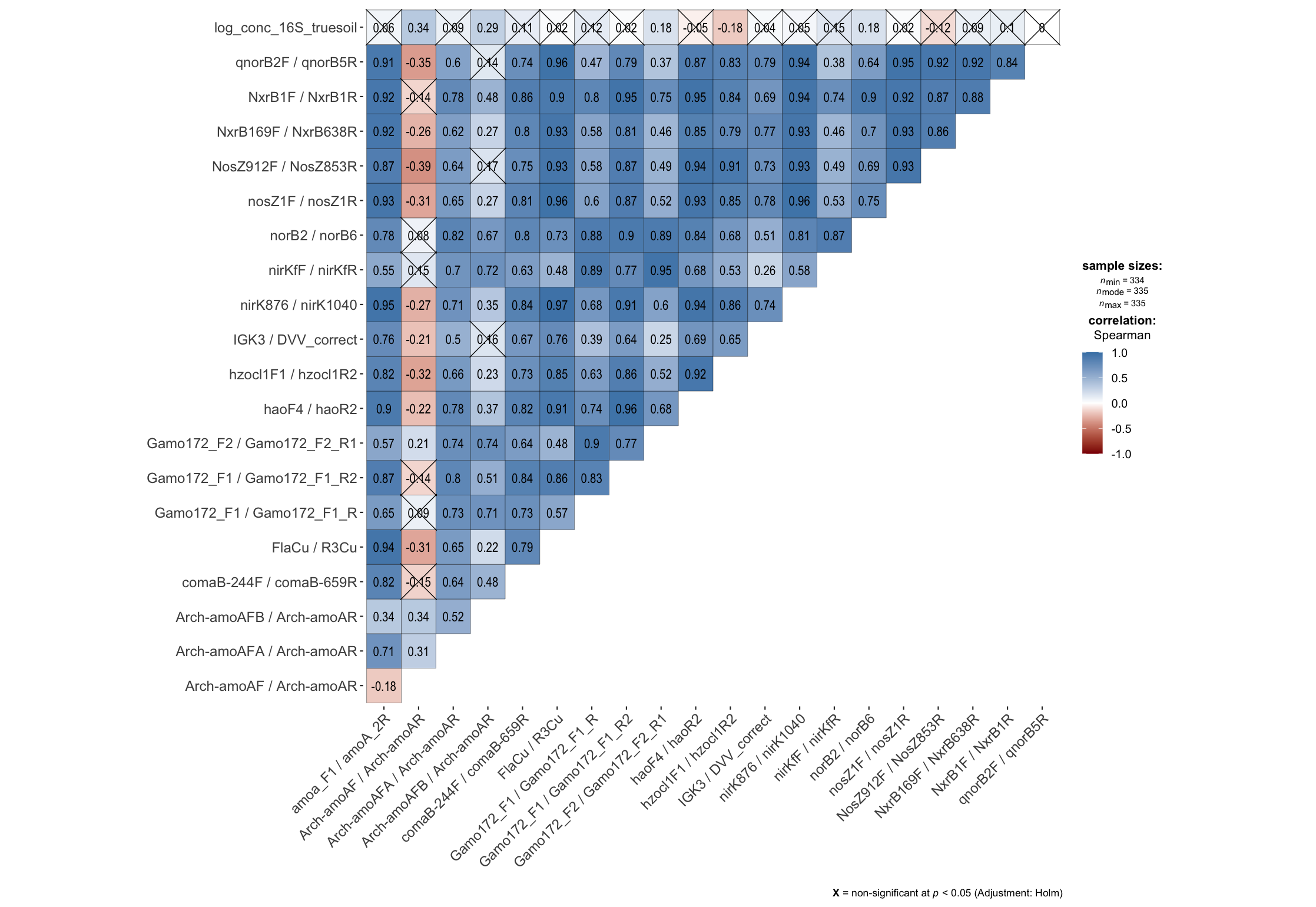

Supplement: S3 Fig — An ‘X’ denotes a non-significant Spearman’s correlation at the level of p = 0.05. Made with the ‘ggstatsplot’ package in R. (TIFF) [file pone.0281442.s004.tiff]

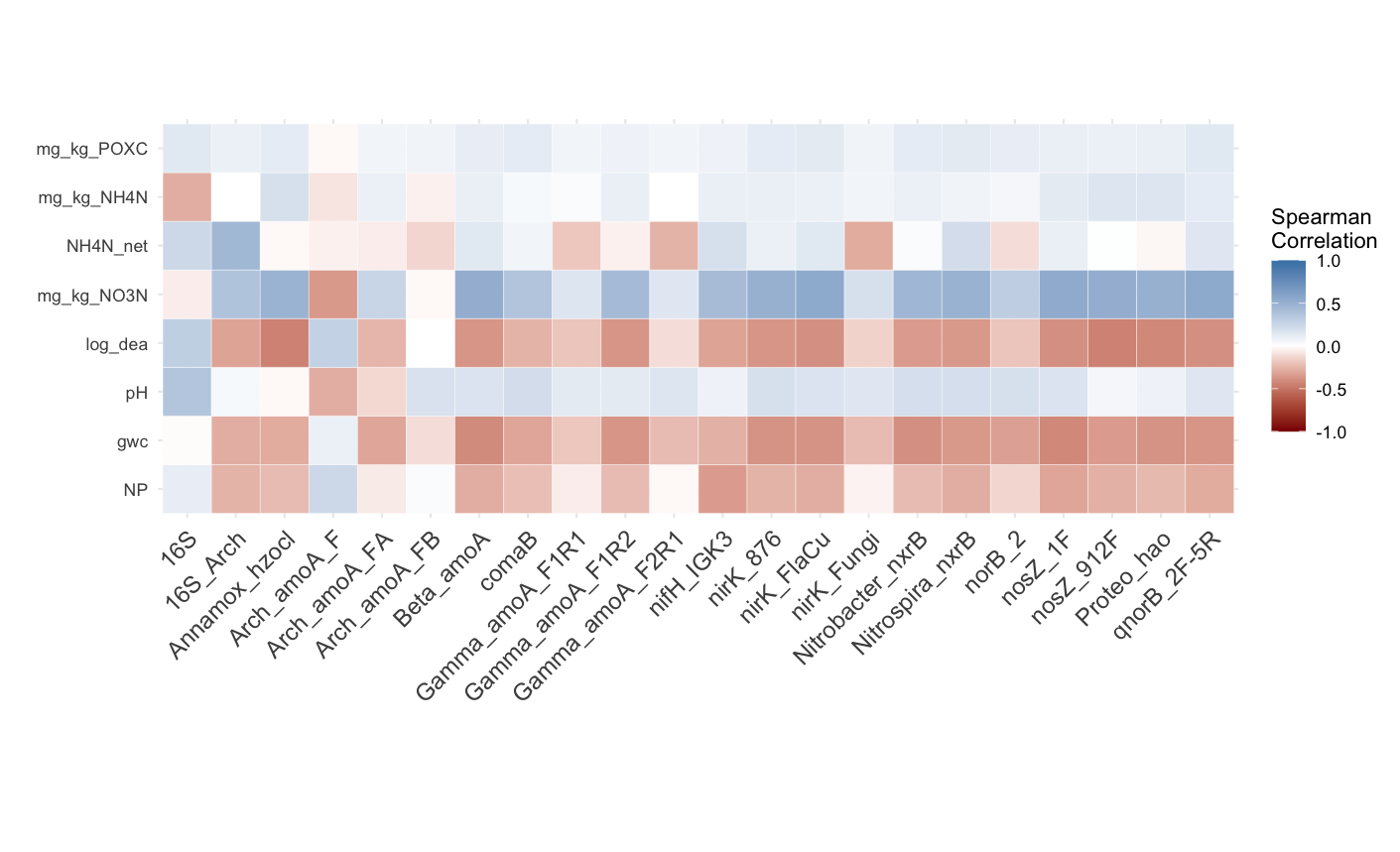

Supplement: S4 Fig — (TIFF) [file pone.0281442.s005.tiff]

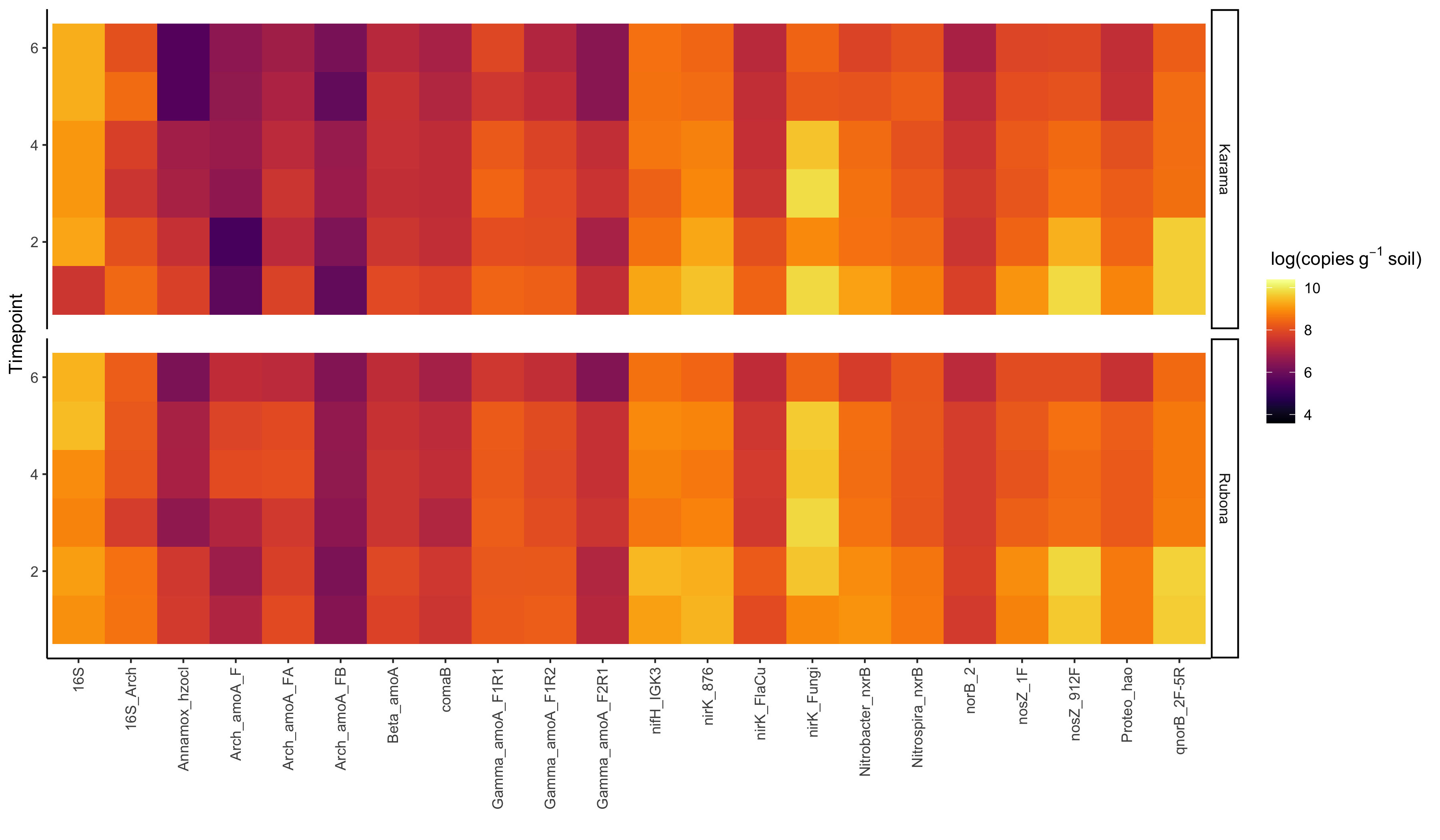

Supplement: S5 Fig — Gene abundances were normalized to log10 copies per gram of soil. (TIFF) [file pone.0281442.s006.tiff]

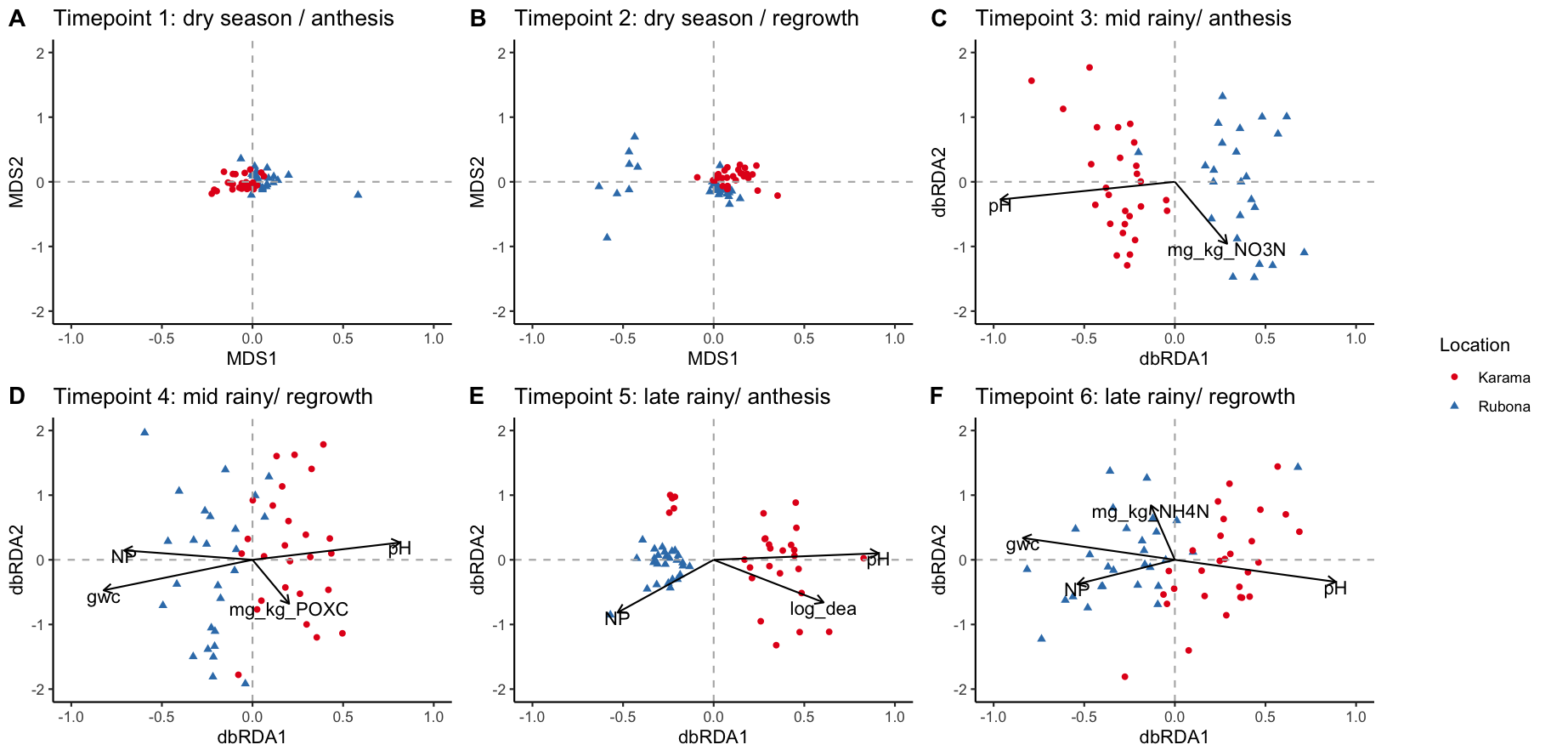

Supplement: S6 Fig — A Spearman’s dissimilarity matrix was used as the input for each dbRDA. (TIFF) [file pone.0281442.s007.tiff]

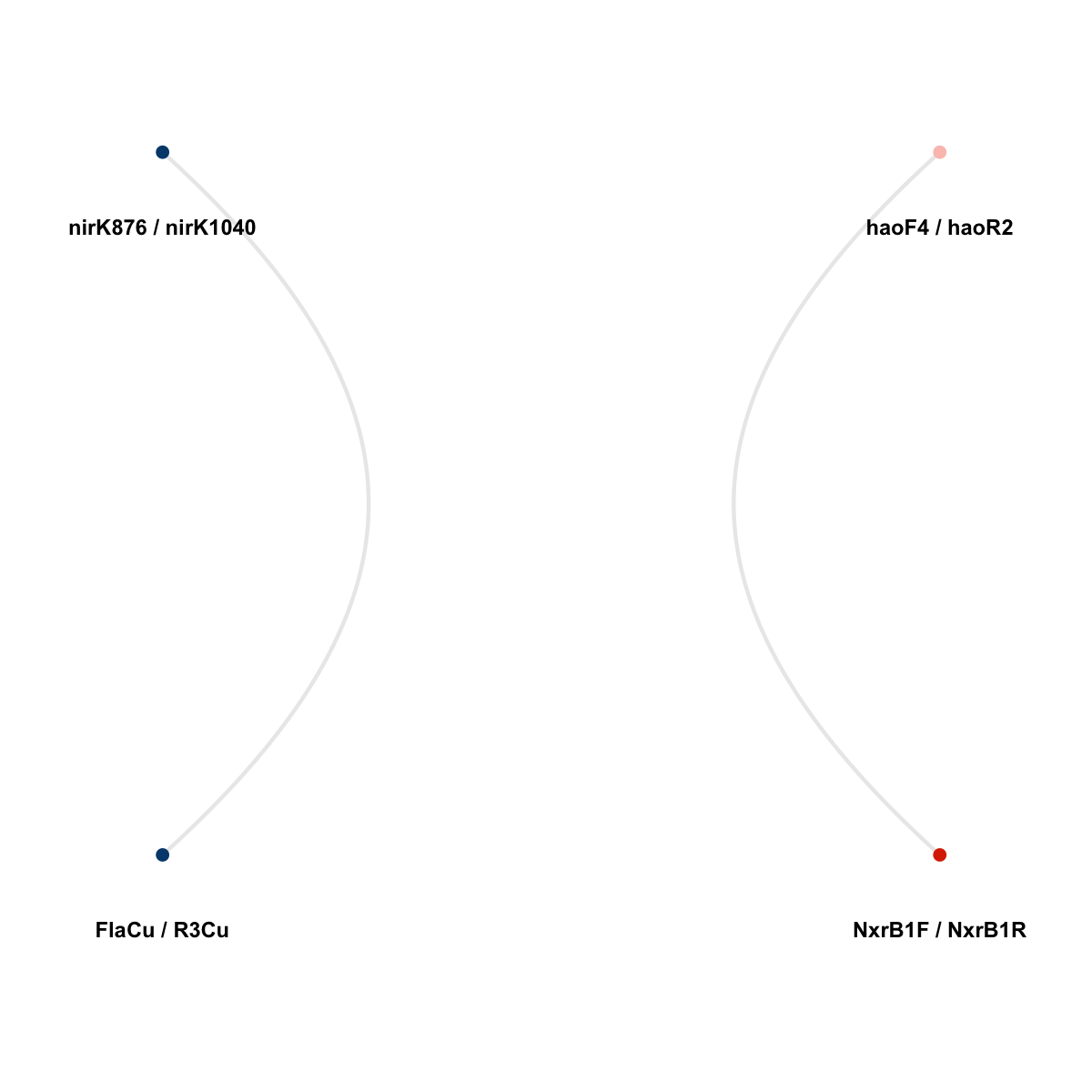

Supplement: S7 Fig — This networks displays N cycle gene co-occurrences that were common across all sampling timepoints and both locations. Nodes representing nitrification gene targets are shown in warm colors, while denitrification gene targets are shown in cool colors. Redundant targets for the same gene and organism were given the same color. (TIFF) [file pone.0281442.s008.tiff]

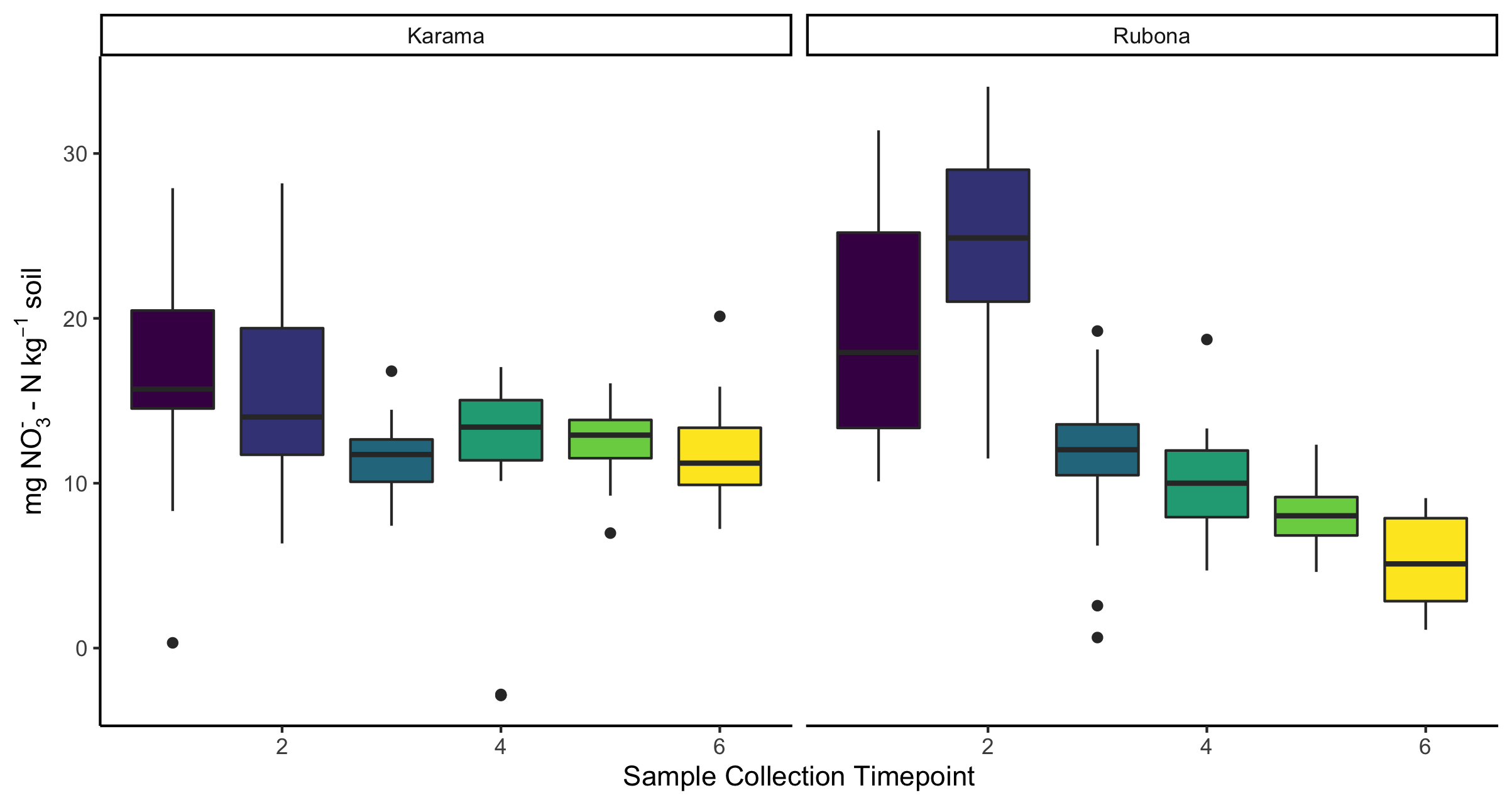

Supplement: S8 Fig — (TIFF) [file pone.0281442.s009.tiff]
