# Supplementary material for: Temporal assessment of N-cycle microbial functions in a tropical agricultural soil using gene co-occurrence networks
Source: PLoS One. 2023 Feb 14;18(2):e0281442. doi: 10.1371/journal.pone.0281442 (PMC9928094; doi:10.1371/journal.pone.0281442)
Supplement: S2 Table — (DOCX) [file pone.0281442.s011.docx]

| **Assay number** | **Assay ID** | **Target** | **Primer name** | **Sequence (5’ > 3’)** | **Used in analysis (Y/N)** | **References** |
| --- | --- | --- | --- | --- | --- | --- |
| 2 | 16S_Arch | Total archaea | Archaea-F KO | CCCTAYGGGGYGCASCAGGC | Y | [1] |
|  |  |  | Archaea-R KO | GCYCYCCCGCCAATTCMTTTA |  |  |
| 3 | Gamma_amoA_F1R1 | AOB (Gamma-proteobacteria) | Gamo172_F1 | GGBGACTGGGAYTTCTGG | Y | [2] |
|  |  |  | Gamo172_F1_R1 | AAARCCCGAGAAGAAMGC |  |  |
| 4 | Gamma_amoA_F1R2 | AOB (Gamma-proteobacteria) | Gamo172_F1 | GGBGACTGGGAYTTCTGG | Y | [2] |
|  |  |  | Gamo172_F1_R2 | AAAACCCGCAAAAAAGGC |  |  |
| 5 | Gamma_amoA_F2_R1 | AOB (Gamma-proteobacteria) | Gamo172_F2 | TGGGATTTCTGGATGGAC | Y | [2] |
|  |  |  | Gamo172_F2_R1 | TGATACGAACGCAGAGAA |  |  |
| 6 | Bac_amoA | AOB (Beta-proteobacteria) | amoA_F1 | GGGGHTTYTACTGGTGGT | Y | [3] |
|  |  |  | amoA_2R | CCCCTCKGSAAAGCCTTCTTC |  |  |
| 7 | Arch_amoA_F | AOA | Arch-amoAF | STAATGGTCTGGCTTAGACG | Y | [4] |
|  |  |  | Arch-amoAR | GCGGCCATCCATCTGTATGT |  |  |
| 8 | Arch_amoA_FA | AOA | Arch-amoAFA | ACACCAGTTTGGYTACCWTCDGC | Y | [5] |
|  |  |  | Arch-amoAR | GCGGCCATCCATCTGTATGT |  |  |
| 9 | Arch_amoA_FB | AOA | Arch-amoAFB | CATCCRATGTGGATTCCATCDTG | Y | [5] |
|  |  |  | Arch-amoAR | GCGGCCATCCATCTGTATGT |  |  |
| 10 |  | AOA | Arch-amoA-for | CTGAYTGGGCYTGGACATC | N | [6] |
|  |  |  | Arch-amoA-rev | TTCTTCTTTGTTGCCCAGTA |  |  |
| 11 | hzocl_1F1 | hzo / anammox_bacteria | hzocl1F1 | TGYAAGACYTGYCAYTGG | Y | [7] |
|  |  |  | hzocl1R2 | ACTCCAGATRTGCTGACC |  |  |
| 12 | Proteo_hao | hao_hdh / proteobacteria_AOB | haoF4 | AYCTKCGCTCRATGGG | Y | [8] |
|  |  |  | haoR2 | GGTTGGTYTTCTGKCCGG |  |  |
| 13 | Annamox_hzocl | hzs / anammox_bacteria | hzsA_1597F | WTYGGKTATCARTATGTAG | N | [2,9] |
|  |  |  | hzsA1857R | AAABGGYGAATCATARTGGC |  |  |
| 14 | Nitrobacter_nxrB | nxrB / proteobacteria_Nitrobacter | NxrB1F | ACGTGGAGACCAAGCCGGG | Y | [10] |
|  |  |  | NxrB1R | CCGTGCTGTTGAYCTCGTTGA |  |  |
| 15 | Nitrospira_nxrB | nxrB / Nitrospirae_Nitrospira | NxrB169F | TACATGTGGTGGAACA | Y |  |
|  |  |  | NxrB638R | CGGTTCTGGTCRATCA |  |  |
| 17 |  | narG / bacteria | narG1960F | AYGTSGGSCARGARAA | Y | [12] |
|  |  |  | narG2650R | TYTCRTACCABGTBGC |  |  |
| 18 |  | nrfA / bacteria | nrfA2aw | CARTGYCAYGTBGARTA | N | [13] |
|  |  |  | nrfAR1 | TWNGGCATRTGRCARTC |  |  |
| 19 |  | napA / bacteria | V66 | TAYTTYYTNHSNAARATHATGTAYGG | N | [14] |
|  |  |  | V67 | DATNGGRTGCATYTCNGCCATRTT |  |  |
| 22 |  | nirS / bacteria | nirSC1F | ATCGTCAACGTCAARGARACVGG | N | [15] |
|  |  |  | nirSC1R | TTCGGGTGCGTCTTSAGAASAG |  |  |
| 23 |  | nirS / bacteria | nirSC2F | TGGAGAACGCCGGNCARGTNTGG | N | [15] |
|  |  |  | nirSC2R | GATGATGTCCACGGCNACRTANGG |  |  |
| 25 | nirK_FlaCu | nirK / bacteria | FlaCu | ATCATGGTSCTGCCGCG | Y | [16] |
|  |  |  | R3Cu | GCCTCGATCAGRTTGTGGTT |  |  |
| 26 | nirK_876 | nirK / bacteria | nirK876 | ATYGGCGGVAYGGCGA | Y | [17] |
|  |  |  | nirK1040 | GCCTCGATCAGRTTRTGGTT |  |  |
| 28 |  | nirK / bacteria | nirKC2F | TGCACATCGCCAACggnatgtwygg | N | [15] |
|  |  |  | nirKC2R | GGCGCGGAAGATGshrtgrtcnac |  |  |
| 31 | norB_2 | norB / denitrifier | norB2 | GACAARHWVTAYTGGTGGT | Y | [18] |
|  |  |  | norB6 | TGCAKSARRCCCCABACBCC |  |  |
| 32 |  | norB / denitrifier | cnorB-2F | GACAAGNNNTACTGGTGGT | N | [19] |
|  |  |  | cnorB-6R | GAANCCCCANACNCCNGC |  |  |
| 33 | qnorB_2F-5R | norB / bacteria | qnorB2F | GGNCAYCARGGNTAYGA | Y | [19] |
|  |  |  | qnorB5R | ACCCANAGRTGNACNACCCACCA |  |  |
| 34 |  | norB / bacteria | qnorB2F | GGNCAYCARGGNTAYGA | N | [19] |
|  |  |  | qnorB7R | GGNGGRTTDATCADGAANCC |  |  |
| 35 | nosZ_1F | nosZ / denitrifier_clade_I | nosZ1F | WCSYTGTTCMTCGACAGCCAG | Y | [20] |
|  |  |  | nosZ1R | ATGTCGATCARCTGVKCRTTYTC |  |  |
| 36 |  | nosZ / denitrifier_clade_I | nosZ-F-1181 | CGCTGTTCITCGACAGYCAG | N | [21] |
|  |  |  | nosZ-R-1880 | ATGTGCAKIGCRTGGCAGAA |  |  |
| 37 |  | nosZ / denitrifier_clade_II | nosZ-II-F | CTIGGICCIYTKCAYAC | N | [22] |
|  |  |  | nosZ-II-R | GCIGARCARAAITCBGTRC |  |  |
| 38 | nosZ_912F | nosZ / denitrifier_clade_II | nosZ912F | CGTCCCCGGCCTCGTGTA | Y | [23] |
|  |  |  | nosZ853R | GAGCAGAAGTTCGTGCAGTAGTAGGG |  |  |
| 39 |  | nifH / bacteria | nifHF | AAAGGYGGWATCGGYAARTCCACCAC | N | [24] |
|  |  |  | nifHR | TTGTTSGCSGCRTACATSGCCATCAT |  |  |
| 40 | nifH_IGK3 | nifH / bacteria | IGK3 | GCIWTHTAYGGIAARGGIGGIATHGGIAA | Y | [25] |
|  |  |  | DVV | ATIGCRAAICCICCRCAIACIACRTC |  |  |
| 41 |  | amoA / comammox | coma-244F | TAYAAYTGGGTSAAYTA | N | [26] |
|  |  |  | coma-659R | ARATCATSGTGCTRTG |  |  |
| 42 | comaB | amoA / comammox | comaB-244F | TAYAAYTGGGTSAAYTA | Y | [26] |
|  |  |  | comaB-659R | ARATCCARACDGTGTG |  |  |
| 43 | nirK_Fungi | nirK / fungi | nirKfF | TACGGGCTCATGTAYGTNSARCC | Y | [26] |
|  |  |  | nirKfR | AGGAATCCCACASCNCCYTTNTC |  |  |

**References**

1. Murakami S, Fujishima K, Tomita M, Kanai A. Metatranscriptomic Analysis of Microbes in an Oceanfront Deep-Subsurface Hot Spring Reveals Novel Small RNAs and Type-Specific tRNA Degradation. Applied and Environmental Microbiology. 2012;78: 1015–1022. doi:10.1128/AEM.06811-11

2. Oshiki M, Segawa T, Ishii S. Nitrogen cycle evaluation (NiCE) chip for simultaneous analysis of multiple N cycleassociated genes. Applied and Environmental Microbiology. 2018;84: 1–15. doi:10.1128/AEM.02615-17

3. Rotthauwe JH, Witzel KP, Liesack W. The ammonia monooxygenase structural gene amoA as a functional marker: molecular fine-scale analysis of natural ammonia-oxidizing populations. Appl Environ Microbiol. 1997;63: 4704–4712. doi:10.1128/aem.63.12.4704-4712.1997

4. Francis CA, Roberts KJ, Beman JM, Santoro AE, Oakley BB. Ubiquity and diversity of ammonia-oxidizing archaea in water columns and sediments of the ocean. Proc Natl Acad Sci U S A. 2005;102: 14683–14688. doi:10.1073/pnas.0506625102

5. Beman JM, Popp BN, Francis CA. Molecular and biogeochemical evidence for ammonia oxidation by marine Crenarchaeota in the Gulf of California. ISME J. 2008;2: 429–441. doi:10.1038/ismej.2007.118

6. Wuchter C, Abbas B, Coolen MJL, Herfort L, van Bleijswijk J, Timmers P, et al. Archaeal nitrification in the ocean. Proc Natl Acad Sci U S A. 2006;103: 12317–12322. doi:10.1073/pnas.0600756103

7. Li M, Hong Y, Klotz MG, Gu J-D. A comparison of primer sets for detecting 16S rRNA and hydrazine oxidoreductase genes of anaerobic ammonium-oxidizing bacteria in marine sediments. Appl Microbiol Biotechnol. 2010;86: 781–790. doi:10.1007/s00253-009-2361-5

8. Schmid MC, Hooper AB, Klotz MG, Woebken D, Lam P, Kuypers MMM, et al. Environmental detection of octahaem cytochrome c hydroxylamine/hydrazine oxidoreductase genes of aerobic and anaerobic ammonium-oxidizing bacteria. Environmental Microbiology. 2008;10: 3140–3149. doi:10.1111/j.1462-2920.2008.01732.x

9. Harhangi HR, Le Roy M, van Alen T, Hu B-L, Groen J, Kartal B, et al. Hydrazine synthase, a unique phylomarker with which to study the presence and biodiversity of anammox bacteria. Appl Environ Microbiol. 2012;78: 752–758. doi:10.1128/AEM.07113-11

10. Vanparys B, Spieck E, Heylen K, Wittebolle L, Geets J, Boon N, et al. The phylogeny of the genus Nitrobacter based on comparative rep-PCR, 16S rRNA and nitrite oxidoreductase gene sequence analysis. Syst Appl Microbiol. 2007;30: 297–308. doi:10.1016/j.syapm.2006.11.006

11. Pester M, Maixner F, Berry D, Rattei T, Koch H, Lücker S, et al. NxrB encoding the beta subunit of nitrite oxidoreductase as functional and phylogenetic marker for nitrite-oxidizing Nitrospira. Environ Microbiol. 2014;16: 3055–3071. doi:10.1111/1462-2920.12300

12. Philippot L, Piutti S, Martin-Laurent F, Hallet S, Germon JC. Molecular analysis of the nitrate-reducing community from unplanted and maize-planted soils. Appl Environ Microbiol. 2002;68: 6121–6128. doi:10.1128/AEM.68.12.6121-6128.2002

13. Welsh A, Chee-Sanford JC, Connor LM, Löffler FE, Sanford RA. Refined NrfA Phylogeny Improves PCR-Based nrfA Gene Detection. Appl Environ Microbiol. 2014;80: 2110–2119. doi:10.1128/AEM.03443-13

14. Flanagan DA, Gregory LG, Carter JP, Karakas-Sen A, Richardson DJ, Spiro S. Detection of genes for periplasmic nitrate reductase in nitrate respiring bacteria and in community DNA. FEMS Microbiol Lett. 1999;177: 263–270. doi:10.1111/j.1574-6968.1999.tb13742.x

15. Wei W, Isobe K, Nishizawa T, Zhu L, Shiratori Y, Ohte N, et al. Higher diversity and abundance of denitrifying microorganisms in environments than considered previously. ISME J. 2015;9: 1954–1965. doi:10.1038/ismej.2015.9

16. Throbäck IN, Enwall K, Jarvis A, Hallin S. Reassessing PCR primers targeting nirS, nirK and nosZ genes for community surveys of denitrifying bacteria with DGGE. FEMS Microbiol Ecol. 2004;49: 401–417. doi:10.1016/j.femsec.2004.04.011

17. Henry S, Baudoin E, López-Gutiérrez JC, Martin-Laurent F, Brauman A, Philippot L. Quantification of denitrifying bacteria in soils by nirK gene targeted real-time PCR. J Microbiol Methods. 2004;59: 327–335. doi:10.1016/j.mimet.2004.07.002

18. Casciotti KL, Ward BB. Phylogenetic analysis of nitric oxide reductase gene homologues from aerobic ammonia-oxidizing bacteria. FEMS Microbiology Ecology. 2005;52: 197–205. doi:10.1016/j.femsec.2004.11.002

19. Braker G, Tiedje JM. Nitric oxide reductase (norB) genes from pure cultures and environmental samples. Appl Environ Microbiol. 2003;69: 3476–3483. doi:10.1128/AEM.69.6.3476-3483.2003

20. Henry S, Bru D, Stres B, Hallet S, Philippot L. Quantitative detection of the nosZ gene, encoding nitrous oxide reductase, and comparison of the abundances of 16S rRNA, narG, nirK, and nosZ genes in soils. Applied and Environmental Microbiology. 2006;72: 5181–5189. doi:10.1128/AEM.00231-06

21. Rich JJ, Heichen RS, Bottomley PJ, Cromack K, Myrold DD. Community Composition and Functioning of Denitrifying Bacteria from Adjacent Meadow and Forest Soils. Applied and Environmental Microbiology. 2003;69: 5974–5982. doi:10.1128/AEM.69.10.5974-5982.2003

22. Jones CM, Graf DRH, Bru D, Philippot L, Hallin S. The unaccounted yet abundant nitrous oxide-reducing microbial community: A potential nitrous oxide sink. ISME Journal. 2013;7: 417–426. doi:10.1038/ismej.2012.125

23. Chee-Sanford JC, Connor L, Krichels A, Yang WH, Sanford RA. Hierarchical detection of diverse Clade II (atypical) nosZ genes using new primer sets for classical- and multiplex PCR array applications. Journal of Microbiological Methods. 2020;172: 105908. doi:10.1016/j.mimet.2020.105908

24. Rösch C, Mergel A, Bothe H. Biodiversity of denitrifying and dinitrogen-fixing bacteria in an acid forest soil. Appl Environ Microbiol. 2002;68: 3818–3829. doi:10.1128/AEM.68.8.3818-3829.2002

25. Gaby JC, Buckley DH. A Comprehensive Evaluation of PCR Primers to Amplify the nifH Gene of Nitrogenase. Balcazar JL, editor. PLoS ONE. 2012;7: e42149. doi:10.1371/journal.pone.0042149

26. Pjevac P, Schauberger C, Poghosyan L, Herbold CW, van Kessel MAHJ, Daebeler A, et al. AmoA-Targeted Polymerase Chain Reaction Primers for the Specific Detection and Quantification of Comammox Nitrospira in the Environment. Frontiers in Microbiology. 2017;8. Available: https://www.frontiersin.org/article/10.3389/fmicb.2017.01508
