# Supplementary material for: Temporal assessment of N-cycle microbial functions in a tropical agricultural soil using gene co-occurrence networks
Source: PLoS One. 2023 Feb 14;18(2):e0281442. doi: 10.1371/journal.pone.0281442 (PMC9928094; doi:10.1371/journal.pone.0281442)
Supplement: S1 Text — (DOCX) [file pone.0281442.s014.docx]

**Supplemental Information**

*Conventional qPCR for 16S rRNA gene*

Conventional quantitative polymerase chain reaction (qPCR) of the bacterial 16S rRNA gene was used to assess DNA extraction quality and test for differences in bacterial abundance among treatments, collection timepoint, and location (Ishii et al., 2014). We used a previously validated protocol for SYBR green qPCR (Fukushima et al, 2003). Prior to running qPCR, we diluted each sample 10-fold using nuclease free water. All procedures were done in a PCR workstation that was sterilized before use for 30 minutes with a UV lamp. All PCR tubes, strips, and plates were similarly UV-irradiated prior to use for 10 minutes in a UV crosslinker. Pipettes, tip boxes, and all other materials were decontaminated with DNAZap (Life Technologies #AM9890).

Each reaction had a volume of 10 µl, which included 1X Sso Advanced Universal SYBR Green Supermix (Bio-Rad Laboratories, CA, USA), 0.5 µM each of forward and reverse primers, and 1 µl of DNA template. At least one negative control consisting of nuclease free water in place of template DNA was included on each plate. Serial dilutions consisting of known quantities of a gBlock standard were used, ranging from 10^0^ to 10^7^ copies/µl.

Conventional qPCR was performed using StepOnePlus Real-Time PCR System (Applied Biosystems, MA, USA) under the following conditions: initial annealing at 98°C for 3 minutes, followed by 40 cycles of 98°C for 10 seconds and 60°C for 30 seconds. Finally, melt curve analysis was performed from 60°C to 95°C. We compiled standard curves for each run and used them to calculate 16S rRNA gene copy number in our unknown samples. Data processing and quality control for conventional 16S rRNA gene qPCR were performed in R (version 4.0.3).

*NiCE chip*

Primer pair mix consisting of a final concentration of 5 µM (10X) of each primer and 1X Tris-EDTA (pH 8.0) was prepared in a 96-well PCR plate and then transferred to a 384-well plate (17.82 ul per well). Aliquot (4.51 µl) of assay mastermix (1.25X SmartChip TB Green Gene Expression Master Mix [Takara Bio]) was subsequently added to each well in the 384-well plate containing the primer mix. This 384-well ‘assay source plate’ was vortexed vigorously for 5 seconds and centrifuged for 5 minutes at 3,320 rpm or until all bubbles were removed. The assay source plate was kept at 4°C in the dark until loading onto the SmartChip Multisample NanoDispenser (MSND).

A 384-well ‘sample source plate’ was similarly prepared using the extracted DNA from our soil samples. We first prepared a sample mastermix which consisted of 1.25X SmartChip TB Green Gene Expression Master Mix and 2.86 µl of DNA sample (or gBlock standards; see below) in 96-well PCR plates. The sample mixture (13.1 µl) were subsequently transferred to the 384-well sample source plate, which was vortexed and centrifuged to remove bubbles as described above. To generate standard curves, gBlock standards of known concentration were serially diluted in a mixture (5 x 10^0^ to 5 x 10^7^ copies/µl) [1] and included in the sample source plate.

Samples and primer mix were dispensed from the 384-well source plates onto the SmartChip using the integrated MSND. The interior of the MSND is sealed and kept at a relative humidity of 30-70% to ensure reproducibility of assay volume and thermal uniformity. After the NanoDispenser completes the transfer of sample and mastermix, the SmartChip was centrifuged at 4°C and kept in the dark until the final transfer into the SmartChip Real-Time PCR cycler. The final reaction volume in each well was 100 nl, consisting of 50 nl each of the sample and assay mixtures.

*SmartChip data processing*

Threshold cycle (C_t_) values were determined for each assay using the SmartChip qPCR software. As described previously, standard curves for each assay were generated by linear regression of the C_t_ values versus the known quantities of gblock template [1,2]. All standard curves had an R^2^ value greater than or equal to 0.90 as part of our quality control pipeline.

Assays for which a standard curve failed to amplify were excluded from analysis. In addition, assays for which >50% of samples failed to amplify or assays that demonstrated strong batch effects between SmartChip runs were also excluded. We defined batch effects as cases in which >60% samples failed to amplify during one SmartChip run but <60% failed to amplify on a different run. Limit of quantification (LOQ) was defined as the lowest concentration of standards that were reliably detected, which ranged from 1.5-4.5 log copies/µl depending on the assay. For assays in which >60% of samples successfully amplified without apparent batch effects, samples with Ct values below the LOQ were given an imputed value of 0.5*LOQ [3].

Raw data processing removed 14 gene targets based on the previously described criteria for missingness or batch effects. Of the remaining assays, 2.3% of our samples failed to amplify despite having at least two biological replicates (same assay, treatment, location, timepoint) with successful amplification. We attributed missing values in these cases to machine error and assigned them a mean Ct value from within their biological replicate.

We normalized our results to a final unit of log copies g^-1^ soil using the following equation that accounts for the dilution factor, DNA extraction volume, and soil gravimetric water content (GWC):

log_10_((copies/µl* 10 * 50 µl)/[0.25-(0.25*GWC)] g soil)

**References**

1. Jang J, Xiong X, Liu C, Yoo K, Ishii S. Invasive earthworms alter forest soil microbiomes and nitrogen cycling. Soil Biol Biochem. 2022;171: 108724. doi:10.1016/j.soilbio.2022.108724

2. Ishii S, Segawa T, Okabe S. Simultaneous Quantification of Multiple Food- and Waterborne Pathogens by Use of Microfluidic Quantitative PCR. Appl Environ Microbiol. 2013;79. doi:10.1128/AEM.00205-13

3. Hites RA. Correcting for Censored Environmental Measurements. Environ Sci Technol. 2019;53: 11059–11060. doi:10.1021/acs.est.9b05042
