# Supplementary material for: Temporal assessment of N-cycle microbial functions in a tropical agricultural soil using gene co-occurrence networks
Source: PLoS One. 2023 Feb 14;18(2):e0281442. doi: 10.1371/journal.pone.0281442 (PMC9928094; doi:10.1371/journal.pone.0281442)
Supplement: Attachment — Submitted filename: Response to Reviewers.docx [file pone.0281442.s015.docx]

**Response to Reviewers**

When submitting your revision, we need you to address these additional requirements.

1. Please ensure that your manuscript meets PLOS ONE's style requirements, including those for file naming. The PLOS ONE style templates can be found at 
https://journals.plos.org/plosone/s/file?id=wjVg/PLOSOne_formatting_sample_main_body.pdf and 
https://journals.plos.org/plosone/s/file?id=ba62/PLOSOne_formatting_sample_title_authors_affiliations.pdf

Thank you, we have reviewed PLOS ONE’s style requirements and updated the file names, author affiliations, and manuscript formatting accordingly.

2. Please include a complete copy of PLOS’ questionnaire on inclusivity in global research in your revised manuscript. Our policy for research in this area aims to improve transparency in the reporting of research performed outside of researchers’ own country or community. The policy applies to researchers who have travelled to a different country to conduct research, research with Indigenous populations or their lands, and research on cultural artefacts. The questionnaire can also be requested at the journal’s discretion for any other submissions, even if these conditions are not met.  Please find more information on the policy and a link to download a blank copy of the questionnaire here: https://journals.plos.org/plosone/s/best-practices-in-research-reporting. Please upload a completed version of your questionnaire as Supporting Information when you resubmit your manuscript.

We have completed the questionnaire and have included it with the manuscript resubmission.

3. We note that the grant information you provided in the ‘Funding Information’ and ‘Financial Disclosure’ sections do not match.

When you resubmit, please ensure that you provide the correct grant numbers for the awards you received for your study in the ‘Funding Information’ section.

We have verified that the grant numbers are correct and updated the Funding Information section in the manuscript. It should be noted that no grant number exists for the University of Minnesota Doctoral Dissertation Fellowship or the CGIAR fund.

4. Thank you for stating the following in your Competing Interests section:  
"The authors declare no competing interests."

Please complete your Competing Interests on the online submission form to state any Competing Interests. If you have no competing interests, please state ""The authors have declared that no competing interests exist."", as detailed online in our guide for authors at http://journals.plos.org/plosone/s/submit-now 
 This information should be included in your cover letter; we will change the online submission form on your behalf.

We have reviewed the online submission form and modified our Competing Interests statement in the manuscript to read “The authors have declared that no competing interests exist.”

5. We note that you have indicated that data from this study are available upon request. PLOS only allows data to be available upon request if there are legal or ethical restrictions on sharing data publicly. For more information on unacceptable data access restrictions, please see http://journals.plos.org/plosone/s/data-availability#loc-unacceptable-data-access-restrictions.

In your revised cover letter, please address the following prompts:

a) If there are ethical or legal restrictions on sharing a de-identified data set, please explain them in detail (e.g., data contain potentially sensitive information, data are owned by a third-party organization, etc.) and who has imposed them (e.g., an ethics committee). Please also provide contact information for a data access committee, ethics committee, or other institutional body to which data requests may be sent.

There are no restrictions on sharing the dataset, as indicated in the revised cover letter.

b) If there are no restrictions, please upload the minimal anonymized data set necessary to replicate your study findings as either Supporting Information files or to a stable, public repository and provide us with the relevant URLs, DOIs, or accession numbers. For a list of acceptable repositories, please see <http://journals.plos.org/plosone/s/data-availability#loc-recommended-repositories>.

The dataset and associated metadata that we used for all analyses and figure generation have been uploaded to a public github repository (<https://www.github.com/schaedem/NiCE_Chip_Paper>). This is now indicated in the cover letter and the Data Availability Statement in the manuscript file.

We will update your Data Availability statement on your behalf to reflect the information you provide.

6. We note that you have included the phrase “data not shown” in your manuscript. Unfortunately, this does not meet our data sharing requirements. PLOS does not permit references to inaccessible data. We require that authors provide all relevant data within the paper, Supporting Information files, or in an acceptable, public repository. Please add a citation to support this phrase or upload the data that corresponds with these findings to a stable repository (such as Figshare or Dryad) and provide and URLs, DOIs, or accession numbers that may be used to access these data. Or, if the data are not a core part of the research being presented in your study, we ask that you remove the phrase that refers to these data.

We have removed the phrase ‘data not shown’ and included an additional supporting figure (S8 Fig) to cite in its place.

7. Please include your full ethics statement in the ‘Methods’ section of your manuscript file. In your statement, please include the full name of the IRB or ethics committee who approved or waived your study, as well as whether or not you obtained informed written or verbal consent. If consent was waived for your study, please include this information in your statement as well.

We did not seek approval from the IRB or other ethics committee because the research is not defined as human subject research according to the DHHS or FDA. Our research involved only soil samples; human subjects were not involved in any way. Our collaborating partner CIAT received a research permit to work in Rwanda, which included a wider ethics review clearance for the project activities.

In our revised manuscript, we have added an additional section in the methods to detail permissions that we received to collect soil samples.

8. Please include captions for your Supporting Information files at the end of your manuscript, and update any in-text citations to match accordingly. Please see our Supporting Information guidelines for more information: <http://journals.plos.org/plosone/s/supporting-information>.

We have included captions for supporting information files at the end of the manuscript, in accordance with journal guidelines.

9. We note that Figure S1 in your submission contain map image which may be copyrighted. All PLOS content is published under the Creative Commons Attribution License (CC BY 4.0), which means that the manuscript, images, and Supporting Information files will be freely available online, and any third party is permitted to access, download, copy, distribute, and use these materials in any way, even commercially, with proper attribution. For these reasons, we cannot publish previously copyrighted maps or satellite images created using proprietary data, such as Google software (Google Maps, Street View, and Earth). For more information, see our copyright guidelines: <http://journals>.plos.org/plosone/s/licenses-and-copyright.
 
We require you to either (1) present written permission from the copyright holder to publish these figures specifically under the CC BY 4.0 license, or (2) remove the figures from your submission:
 
a. You may seek permission from the original copyright holder of Figure S1 to publish the content specifically under the CC BY 4.0 license.  
 
We recommend that you contact the original copyright holder with the Content Permission Form (<http://journals>.plos.org/plosone/s/file?id=7c09/content-permission-form.pdf) and the following text:
“I request permission for the open-access journal PLOS ONE to publish XXX under the Creative Commons Attribution License (CCAL) CC BY 4.0 (<http://creativecommons>.org/licenses/by/4.0/). Please be aware that this license allows unrestricted use and distribution, even commercially, by third parties. Please reply and provide explicit written permission to publish XXX under a CC BY license and complete the attached form.”
 
Please upload the completed Content Permission Form or other proof of granted permissions as an “”Other”” file with your submission.
 
In the figure caption of the copyrighted figure, please include the following text: “Reprinted from [ref] under a CC BY license, with permission from [name of publisher], original copyright [original copyright year].”
 
b. If you are unable to obtain permission from the original copyright holder to publish these figures under the CC BY 4.0 license or if the copyright holder’s requirements are incompatible with the CC BY 4.0 license, please either i) remove the figure or ii) supply a replacement figure that complies with the CC BY 4.0 license. Please check copyright information on all replacement figures and update the figure caption with source information. If applicable, please specify in the figure caption text when a figure is similar but not identical to the original image and is therefore for illustrative purposes only.
The following resources for replacing copyrighted map figures may be helpful:
 
USGS National Map Viewer (public domain): <http://viewer>.nationalmap.gov/viewer/
The Gateway to Astronaut Photography of Earth (public domain): http://eol.jsc.nasa.gov/sseop/clickmap/
Maps at the CIA (public domain): <https://www>.cia.gov/library/publications/the-world-factbook/index.html and <https://www>.cia.gov/library/publications/cia-maps-publications/index.html
NASA Earth Observatory (public domain): <http://earthobservatory>.nasa.gov/
Landsat: <http://landsat>.visibleearth.nasa.gov/
USGS EROS (Earth Resources Observatory and Science (EROS) Center) (public domain): <http://eros>.usgs.gov/#
Natural Earth (public domain): <http://www>.naturalearthdata.com/

Figure S1 was generated using a publicly available database (Natural Earth). This information is now included in the figure caption for S1.

5. Review Comments to the Author

Reviewer #1: Full treatment details need to be given like no. of treatments, names of forage grasses, legumes.

A full description of the forage treatments is now given in lines 206-210.

Results section needs to be elaborated by mentioning keystone genes identified for nitrogen cycle processes.

Thank you for this suggestion. We have now included an expanded description of keystone genes in the results section (lines 506-515).

Figures are not clearly visible, hence prepare high resolution figures for clear visibility.

We have improved the resolution quality of figure 4 and verified that all figures have a minimum resolution of 300 dpi. All figures have now been uploaded to the PACE digital diagnostic tool to verify compliance with PLOS publishing standards.

Table 3 is missing.

Thank you for noticing this error. This was a typo as we do not have a table 3. The text now references S4 Table (line 502).

At some places typo errors are there like copes for copies, thoroughly check the entire manuscript.
Supplemental table 3 and 4 are not cited in the text.

We have scanned the document and corrected any such grammatical or spelling errors. Supplemental tables 3 & 4 are now referenced in the manuscript (lines 406 and 502).

Reference style is not uniform, hence maintain uniformity. In requirement of the above, the manuscript needs major revision.

We have scanned the manuscript and references section and made corrections as needed to ensure uniformity.

Reviewer #2: This manuscript aims to elucidate and characterize temporal changes in nitrogen cycle functional genes, which play a vital role in connecting N cycle functions in agricultural fields, using new technique of HT-qPCR, which is a promising low-cost tool to understand the functional potential of a microbial community. The results are convincing, experiments were properly designed and the findings have important implications towards the change in microbial gene functions related to N-cycle, which is most responsible for availability of nitrogen for crop growth. As the nitrogen is the most crucial element for crops especially in tropical agroecosystems, which is highly managed manmade ecosystem.

Overall, the manuscript is fit for publication. Result portions are written well except at some places written in such that it is difficult to understand the outcome. Result should more clearly written.

Thank you for this feedback. We have added or altered text in the Results section to improve clarity. For instance, we include an expanded description of keystone genes (lines 506-515) and attempted to clarify terminology in several other places.

Conclusions should be written in more elaborate manner. In conclusions, mention in detail about novelty of this work compared to what has already been published to emphasize on the impact of this study.

Thank you for this suggestion. We have added text in the conclusion section to underscore the impact and novelty of this work (lines 713-715 & 719-722). We have also expanded our Discussion section in certain areas to elaborate on our interpretation of the findings. For instance, refer to added text in lines 555-557, 643-647, 651-662, and 695-697. We are happy to make additional changes in this regard if there are specific places requiring further elaboration.
